# Synthesis and Pharmacological Evaluation of Novel Silodosin-Based Arylsulfonamide Derivatives as α_1A_/α_1D_-Adrenergic Receptor Antagonist with Potential Uroselective Profile

**DOI:** 10.3390/molecules23092175

**Published:** 2018-08-29

**Authors:** Vittorio Canale, Aleksandra Rak, Magdalena Kotańska, Joanna Knutelska, Agata Siwek, Marek Bednarski, Leszek Nowiński, Małgorzata Zygmunt, Paulina Koczurkiewicz, Elżbieta Pękala, Jacek Sapa, Paweł Zajdel

**Affiliations:** 1 Department of Medicinal Chemistry, Faculty of Pharmacy, Jagiellonian University Medical College, 9 Medyczna Street, 30-688 Krakow, Poland; vittorio.canale@uj.edu.pl (V.C.); pawel.zajdel@uj.edu.pl (P.Z.); 2 Department of Pharmacological Screening, Faculty of Pharmacy, Jagiellonian University Medical College, 9 Medyczna Street, 30-688 Krakow, Poland; arakus1987@gmail.com (A.R.); magda.dudek@uj.edu.pl (M.K.); joanna.1.knutelska@uj.edu.pl (J.K.); marek.bednarski@uj.edu.pl (M.B.); leszek.nowinski@uj.edu.pl (L.N.); malgorzata.zygmunt@uj.edu.pl (M.Z.); 3 Department of Pharmacobiology, Faculty of Pharmacy, Jagiellonian University Medical College, 9 Medyczna Street, 30-688 Krakow, Poland; agat.siwek@uj.edu.pl; 4 Department of Pharmaceutical Biochemistry, Faculty of Pharmacy, Jagiellonian University Medical College, 9 Medyczna Street, 30-688 Krakow, Poland; paulina.koczurkiewicz@uj.edu.pl (P.K.); elzbieta.pekala@uj.edu.pl (E.P.)

**Keywords:** arylsulfonamides of alicyclic amines, α_1_-adrenoceptor antagonists, α_1A/B/D_ receptor selectivity, silodosin, tamsulosin, uroselective activity, benign prostatic hyperplasia

## Abstract

Benign prostatic hyperplasia (BPH) is the most common male clinical problem impacting the quality of life of older men. Clinical studies have indicated that the inhibition of α_1A_-/α_1D_ adrenoceptors might offer effective therapy in lower urinary tract symptoms. Herein, a limited series of arylsulfonamide derivatives of (aryloxy)ethyl alicyclic amines was designed, synthesized, and biologically evaluated as potent α_1_-adrenoceptor antagonists with uroselective profile. Among them, compound **9** (3-chloro-2-fluoro-*N*-([1-(2-(2-(2,2,2-trifluoroethoxy)phenoxy]ethyl)piperidin-4-yl)methyl)benzenesulfonamide) behaved as an α_1A_-/α_1D_-adrenoceptor antagonist (*K*_i_(α_1_) = 50 nM, EC_50_(α_1A_) = 0.8 nM, EC_50_(α_1D_) = 1.1 nM), displayed selectivity over α_2_-adrenoceptors (*K*_i_(α_2_) = 858 nM), and a 5-fold functional preference over the α_1B_ subtype. Compound **9** showed adequate metabolic stability in rat-liver microsome assay similar to the reference drug tamsulosin (Cl_int_ = 67 and 41 µL/min/mg, respectively). Compound **9** did not decrease systolic and diastolic blood pressure in normotensive anesthetized rats in the dose of 2 mg/kg, *i.v*. These data support development of uroselective agents in the group of arylsulfonamides of alicyclic amines with potential efficacy in the treatment of lower urinary tract symptoms associated to benign prostatic hyperplasia.

## 1. Introduction

α_1_-Adrenergic receptors (α_1_-ARs) belong to the G-protein-coupled receptor superfamily. They generally mediate their actions through G_q/11_ proteins, which stimulate the activation of phospholipase C, via generation of the inositol triphosphate and diacylglycerol, liberation of calcium from the endoplasmic reticulum, and/or activation of genes. To date, three subtypes of α_1_-AR, i.e., α_1A_, α_1B_, and α_1D_ have been identified in human tissues [1]. Although these subtypes display high structural homology, they differ in biological structure, tissue distributions, and pharmacological actions [2]. Several studies revealed that α_1_-AR subtypes are highly expressed in blood vessels—mainly α_1B_-ARs, in the urogenital area (prostate, urethra, bladder, ureter)—mainly α_1A_ and α_1D_-ARs, and central nervous system [3]. α_1_-ARs play an important role in the pathogenesis of hypertension and benign prostatic hyperplasia (BPH) [4,5].

An increased α_1_-adrenergic prostate smooth muscle tone together with enhanced prostate volume are recognized causes of the disease [6]. BPH clinically manifests with lower urinary tract symptoms (LUTS) as storage (irritative) symptoms (nocturia, urgency, incontinence, altered bladder sensations, increased frequency) or obstructive (voiding) symptoms (hesitancy, slow stream, intermittency, splitting, straining, terminal dribble) [7]. Some of them commonly occur secondary to obstructive symptoms, and result from exaggerated, spontaneous detrusor contractions (known as bladder overactivity) [7,8]. BPH affects the majority of men with increasing frequency as they get older [9]. LUTS, if left untreated, result in significant impairment of quality of life and lead to long-term complications, such as recurrent urinary tract infections or renal insufficiency [10].

Despite several classes of BPH medications available, studies have shown that α_1_-adrenolitics are considered as the first-line drug treatment [11]. It has been suggested that enhanced, three-to-nine-fold greater expression of α_1A_- and α_1D_-ARs in an enlarged prostate and bladder neck, comparing to healthy tissue, remains in strong contribution with LUTS occurrence [12]. Consequently, an α_1A_- and α_1D_-AR blockade relieves obstructive and voiding symptoms by relaxation of the smooth muscle in the prostate and bladder detrusor, respectively [13]. 

**Figure 1:**
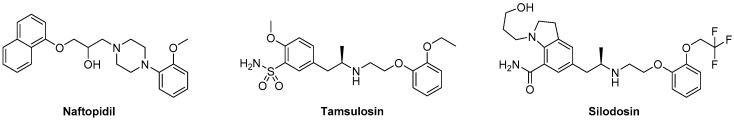
Chemical structures of selective α_1A_- and α_1D_-AR antagonists.

In contrast, a blockade of α_1B_-ARs, which are predominantly expressed in vascular smooth muscle [14], results in vasodilation of blood vessels leading to cardiovascular side effects, especially orthostatic hypotension [15]. The old α_1_-adrenolitics, bearing quinazoline scaffold, i.e., doxazosin or terazosin, display nonspecific interaction with all α_1_-AR subtypes [5]. On the other hand, naftopidil, tamsulosin, and silodosin (Figure 1), displaying relatively high α_1A_- and α_1D_-AR subtype selectivity, effectively relieve symptoms related to BHP/LUTS disease without undesirable side effects on blood pressure [16,17,18]. 

**Figure 2:**
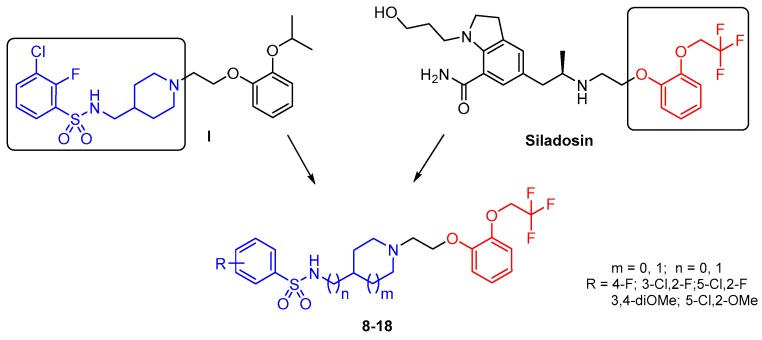
Design strategy for arylsulfonamide derivatives of alicyclic amines as silodosin analogs.

Integrating a concept of arylpiperazine biomimetics recently adapted for development of selective and potent 5-HT_7_R antagonists [19], we explored the common chemical space with tamsulosin to propose modifications leading to increased α_1A_-AR properties. Associating arylsulfonamide and aryloxylalkyl fragments identified compound **I**, which behaved as an α_1A_-AR antagonist and displayed a moderate selectivity receptor profile over α_1B_-AR subtype [20]. In an attempt to further increase the uroselective profile, a limited series of compounds integrating silodosin-derived chemical scaffold was designed (Figure 2). Selection of the central amine core (4-aminomethyl-piperidine and 3-amino-pyrrolidine), as well as a kind of substituent at the arylsulfonamide moiety, was based on our previously reported data presenting their preference for α_1A_-AR over 5-HT_1A_, and 5-HT_7_R [20,21,22]. 

All the synthesized derivatives were *in vitro* evaluated to assess their affinity for α_1_-AR and selectivity over α_2_-AR subtypes. Then, antagonist properties of selected derivatives against α_1A_-, α_1B_-, and α_1D_-AR subtypes were determined in cellular functional assays. The most representative compounds with uroselective functional profile were submitted under extended *in vitro* screening towards off-targets responsible for potential side effects, and were evaluated in metabolic stability in *in vitro* assay to assess their susceptibility to biotransformation. Finally, selected compounds were administered to normotensive anesthetized rats to evaluate their effects on blood pressure as a measure of their potential *in vivo* uroselectivity and to exclude hypotensive effects unfavorable to the treatment of lower urinary tract symptoms associated to benign prostatic hyperplasia.

## 2. Chemistry

**Scheme 1:**
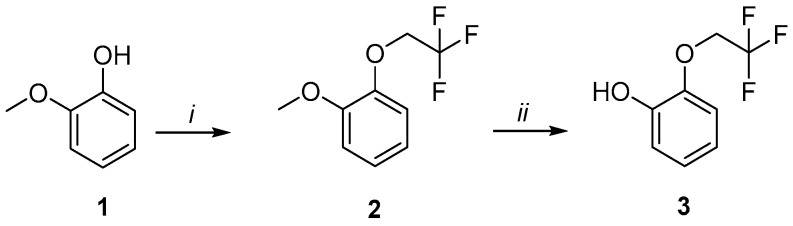
Synthesis of phenol **3**. Reagents and conditions: (*i*) 2-iodo-1,1,1-trifluoroethane, K_2_CO_3_, KI, DMF, 90 °C, 24 h; (*ii*) BBr_3_, CH_2_Cl_2_ anh, 0 °C → r.t., 2 h.

**Scheme 2:**
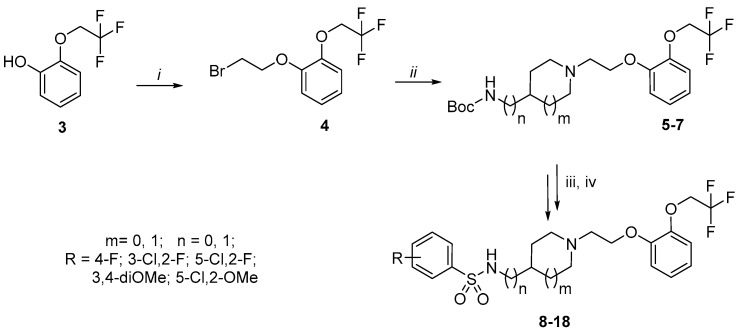
Synthesis of silodosin analogs **8**–**18**. Reagents and conditions: (*i*) 1,2-dibromoethane, K_2_CO_3_, KI, (CH_3_)_2_CO, 60 °C, 48 h; (*ii*) alicyclic amine, K_2_CO_3_, KI, (CH_3_)_2_CO, 60 °C, 24 h; (*iii*) TFA/CH_2_Cl_2_ (80/20; *v*/*v*), r.t., 2 h; (*iv*) arylsulfonyl chloride, TEA, CH_2_Cl_2_, 0 °C, 2–6 h.

The multistep protocol for synthesis of compounds **8**–**18** in outlined in Scheme 1 and Scheme 2. Initially, (2,2,2-trifluoroethoxy)phenol (**3**) was synthesized by alkylation of commercially available guaiacol **1**, followed by demethylation of intermediate **2** in the presence of boron tribromide (Scheme 1).

Next, the alkylation of phenol **3** under biphasic conditions yielded the corresponding (aryloxy)ethyl bromide **4**. Subsequently, this alkylating agent reacted with selected Boc-protected alicyclic amines (4-aminomethyl-piperidine, *R*-3-amino-pyrrolidine, and *S*-3-amino-pyrrolidine), giving intermediates **5**–**7**. Removal of the protecting group, followed by the treatment with selected arylsulfonyl chloride, yielded final arylsulfonamide derivatives **8**–**18**.

## 3. *In Vitro* Experiments

### 3.1. Radioligand Binding and Functional Evaluation

The pharmacological profile of the new compounds was assessed in radioligand-binding assays as the ability to displace [^3^H]-Prazosin or [^3^H]-Clonidine from α_1_- and α_2_-ARs, respectively, on rat cerebral cortex [23]. The inhibition constants (*K*_i_) were calculated from the Cheng-Prusoff equation [24]. 

The intrinsic activity at α_1A_-ARs of the selected compounds was assessed by fluorescence detection (Invitrogen, Thermo Fisher Scientific, Carlsbad, CA, USA) of β-lactamase reporter genes using a FRET-enabled substrate. The intrinsic activity at α_1B_-ARs and α_1D_-ARs was determined by luminescence detection (PerkinElmer, Zaventem, Belgium) of calcium mobilization using the recombinant-expressed jellyfish photoprotein (Aequorin).

The most representative compounds, **9** and **10,** with the highest functional selectivity were further tested to determine the affinity for 5-HT_1A_ and 5-HT_7_Rs in screening radioligand-binding studies using [^3^H]-8-Hydroxy-2-(dipropylamino)tetralin ([^3^H]-8-OH-DPAT) and [^3^H]-Lysergic acid diethylamide ([^3^H]-LSD), respectively. Experiments were performed using membranes from CHO-K1 cells stably transfected with the human 5-HT_1A_ and 5-HT_7_Rs according to the methods previously described [25].

Finally, the percentage of inhibition for selected compounds **9** and **10** for off-target histaminic H_1_R, muscarinic M_1_R, adrenergic β_1_-AR and potassium ion channel *h*ERG were assessed at Eurofins (Celle-Lévescault, France) according to the procedure online at www.eurofins.com [26].

### 3.2. Metabolic Stability

*In vitro* biotransformation assays of selected compounds **9** and **10** were performed using rat-liver microsomes (RLM), potassium-phosphate buffer, NADPH-regenerating system (NADP, glucose-6-phosphate, glucose-6-phosphate dehydrogenase), and levallorphan as internal standard, according to the previously published procedure [27]. Compound **I** and the drug tamsulosin were used as reference standard. UPLC/MS analysis (Waters Corporation, Milford, MA, USA) was performed to determine the quantity of the starting material left in solution. The *in vitro* half-time (t_1/2_) for test compounds was determined from the slope of the linear regression of ln % parent compound remaining versus incubation time. The calculated t_1/2_ was incorporated into the following equation to obtain intrinsic clearance: (Cl_int_) = (volume of incubation [µL]/protein in the incubation [mg]) × 0.693/t_1/2_.

## 4. *In Vivo* Pharmacology

Compounds **9** and **10**, which displayed the highest α_1B_/α_1A_ selectivity profile, were selected for *in vivo* evaluation to determine their influence on blood pressure of normotensive anaesthetized rats after acute administration at single dose of 2 mg/kg (*i.v*.). The experiments were performed to our previously reported method.

## 5. Results and Discussion

All synthesized compounds were *in vitro* evaluated in binding assays for their affinity for α_1_-AR and selectivity over α_2_-AR subtype. Compounds showed high-to-moderate affinity for α_1_-ARs (*K*_i_ = 19–171 nM), and low-to-moderate selectivity over α_2_-AR subtype (Table 1). Analysis of the influence of substituent in position-2 at the aryloxy fragment showed that an increase of its volume by replacing the isopropoxy group present in compound **I** and **II** with the 2,2,2-trifluoroethoxy one (present in a new series) only slightly increased the affinity for α_1_-ARs (**I** *vs*. **9**, **II** *vs*. **16**).

In line with our previous results [20], the 4-aminomethylpiperidine core was more favorable for the binding at α_1_-AR than 3-aminopyrrolidine one (**8** *vs*. **13** and **14**, **12** *vs*. **17** and **18**). Although among compounds with 3-aminopyrrolidine no stereochemical preference towards α_1_-AR was observed, the *S* enantiomers showed higher selectivity over α_2_-AR than their *R* counterparts (**13** *vs*. **14**, **15** *vs*. **16**, **17** *vs*. **18**).

Further modifications involved the introduction of different electron-withdrawing or electron-donating substituents at the phenyl ring of sulfonamide moiety. A fluorine atom in 4-position was sufficient for obtaining a potent α_1_-AR ligand **8** (*K*_i_ = 20 nM) among the 4-aminomethyl-piperidine subset, but did not significantly improve the affinity of pyrrolidine derivatives **13** and **14** for α_1_-AR (*K*_i_ = 188 and 134 nM, respectively). Interestingly, the presence of the 4-F substituent in both series led to derivatives with the highest selectivity over the α_2_-AR subtype (S_α2/α1_ ≥ 46). An introduction of two halogen substituents did not affect the affinity for α_1_-AR while decreasing the selectivity over the α_2_-AR subtype (**8** *vs*. **9** and **10**, **13** *vs*. **15**, and **14** *vs*. **16**). Replacing one of the halogen substituents (e.g., 2-F) in compound **10**, with an electron-donating substituent as the 2-methoxy, up to 4-fold reduced both affinity for α_1_-AR and selectivity over α_2_-AR (**10** *vs*. **11**). Finally, compounds **12**, **17**, and **18,** with two methoxy groups in 3,4-position at the phenyl ring of sulfonamide moiety, showed higher affinity for α_1_-AR than the 4-F direct analogs (**8** *vs*. **12**, **13** *vs*. **17,** and **14** *vs*. **18**); however, this modification decreased the selectivity over α_2_-AR subtype. Selected compounds with the highest affinity for α_1_-ARs (*K*_i_ ≤ 50 nM) and selectivity ratio, which equals >15-fold over α_2_-AR subtype, behaved as potent antagonists at α_1A_-, α_1B_-, and α_1D_-ARs in *in-vitro* functional tests (Table 2). Compounds **8**, **9,** and **10** were classified as more potent antagonists than previously reported compound **I** at all tested α_1A_-, α_1B_-, and α_1D_-ARs. Compounds **9** and **10**, bearing two halogen atoms in *ortho* and *meta* position (i.e., 3-Cl,2-F and 5-Cl,2-F) at the phenyl ring of sulfonamide moiety displayed the highest α_1B_/α_1A_ selectivity ratio. An introduction of the strong electron-donating substituent in *meta* and *para* position (e.g., 3,4-diOMe), switched the functional-selectivity profile of compound **12**, which behaved as a selective α_1B_-AR antagonist (IC_50_ = 0.022 nM).

It is well known that a blockade of α_1A_- and α_1D_-ARs relaxes the enhanced prostate and bladder detrusor smooth muscle tone, whereas α_1B_-AR antagonism is involved in blood-pressure regulation. Normal detrusor, obtained from surgical patients, expresses predominantly α_1D_-ARs. Some pharmacological experiments revealed that highly selective α_1A_-AR antagonists are effective in relaxing prostate smooth muscle and therefore improving urine flow in men in this area. However, relaxation of smooth muscle of the prostate alone does not alter reported LUTS scores in men with BPH. Reduction of these symptoms is reported only when pharmacotherapy includes drugs with both α_1A_- and α_1D_-AR antagonistic activity. Such activity improves bladder-based symptoms in humans and is used in LUTS pharmacotherapy [13]. Compounds **8**, **9**, **10**, and **12** in the intrinsic activity studies showed strong antagonistic properties against the α_1D_-AR subtype with EC_50_ in the range of 1.1 to 2.7 nM. However, among the tested compounds, only **9** and **10** showed a similar inhibitory effect on intrinsic signal transduction in cells with stable expression of human α_1A_- and α_1D_-ARs.

Some pieces of evidence suggest an involvement of serotonin 5-HT_1A_ and 5-HT_7_Rs in regulation of rodent bladder and urethral-sphincter contractions in both in *in vitro* and *in vivo* models [28,29]. Thus, 5-HT_1A_ and 5-HT_7_R ligands may be regarded as adjunctive agents in alleviating LUTS associated to BPH. Compounds **9** and **10** displayed high-to-moderate affinity for 5-HT_1A_ and 5-HT_7_Rs (Table 3).

The same compounds were further evaluated for their affinity for “off-target” receptor panels at Eurofins Cerep, and displayed weak affinity for histamine H_1_, muscarinic M_1_, adrenergic β_1_, and *h*ERG channels (<50% @ 1 μM) [26]. These may suggest a low risk of compounds to evoke undesirable cardiovascular or CNS side effects. An initial assessment of the metabolic fate in liver was subsequently performed in an *in-vitro* RLM model. Compounds **9** and **10** showed relatively low clearances (Cl_int_ = 67 and 91.7 μL/min/mg, respectively, Table 4), with values similar to those of reference compound **I** and the drug tamsulosin (Cl_in__t_ = 87 and 41 μL/min/mg, respectively). The values of internal clearance calculated for the tested compounds are in line with the value of clearance of reference drugs (i.e., propranolol, verapamil) reported in the literature [30,31]. 

Identified compounds **9** and **10** with favorable α_1B_/α_1D_ profile and acceptable metabolic stability were selected for *in vivo* tests to evaluate their influence on blood pressure. Hypotensive activity was determined after one time *i.v.* administration to normotensive anaesthetized rats at single doses of 2 mg/kg according to our previously reported method [32]. 

Compound **9** given at the dose of 2 mg/kg decreased SBP about 5.9–10.9 mmHg (5.1–9.4%), and DBP about 2–5.3 mmHg (2.4–6.5%) insignificantly.

**Figure 3:**
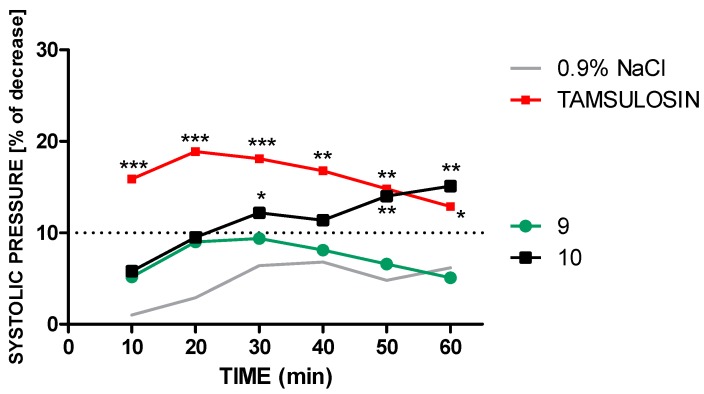
An influence of tamsulosin and compounds **9** and **10**, given in a dose of 2 mg/kg (*i.v.*), on systolic pressure in anaesthetized rats. Significant to control group (0.9% NaCl): * *p* < 0.05, ** *p* < 0.02, *** *p* < 0.01.

**Figure 4:**
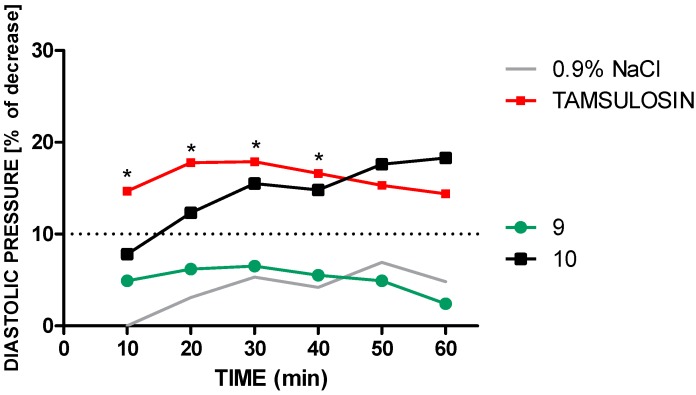
An influence of tamsulosin and compounds **9** and **10**, given in a dose of 2 mg/kg (*i.v.*), on diastolic pressure in anaesthetized rats. Significant to control group (0.9% NaCl): * *p* < 0.05.

Compound **10** in a dose of 2 mg/kg reduced both, SBP about 7–18 mmHg (5.8–15.1%), and DBP about 7–16.4 mmHg (7.8–18.3%). A statistically significant drop in systolic blood pressure was observed from 30 min after administration (Figure 3 and Figure 4).

In contrast, the highly α_1A_-AR-selective drug tamsulosin administered intravenously at a dose of 2 mg/kg decreased SBP 16.2–23.7 mmHg (12.9–18.9%) and the DBP about 13.3–16.6 mmHg (14.4–17.9%) significantly through whole period of observation (Figure 3 and Figure 4).

It thus seems that compound **9** revealed a potential uroselective profile, comparable to tamsulosin, without evoking hypotension as a side effect. These data warrant further investigation of compound **9** in *ex vivo* preclinical models of BPH disease.

## 6. Conclusions

By combining the 2-(2,2,2-trifluoroethoxy)phenoxy fragment of silodosin with an alicyclic amine core functionalized with arylsulfonamide moiety, derived from previously reported compound **I**, we designed and synthesized a new series of arylsulfonamides of (aryloxy)ethyl pyrrolidines and piperidines as α_1_-AR antagonists. Structure–activity relationship studies revealed that the 4-aminomethylpiperidine core was preferential for binding with the α_1_-AR over the 3-aminopyrrolidine analog. Additionally, a kind of substituent at the phenyl ring of sulfonamide significantly impacted the selectivity of evaluated compounds over α_1B_- and α_2_-AR subtypes. The study allowed the identification of compound **9** as a potent and metabolically stable α_1A_-AR antagonist with improved α_1B_/α_1A_ selectivity ratio, comparing with previously reported series. Moreover, compound **9** showed α_1D_-AR antagonistic activity that may be beneficial in terms of LUTS therapy. In contrast to the reference drug tamsulosin, the tested compound did not decrease blood-pressure parameters after acute administration at the dose of 2 mg/kg (*i.v.*) in rats. Preliminary data for compound **9** are promising enough to warrant its further detailed mechanistic studies as a potential uroselective α_1A_- and α_1D_-AR antagonist in the treatment of lower urinary tract symptoms associated with benign prostatic hyperplasia.

## 7. Experimental

### 7.1. Chemistry

#### 7.1.1. General Chemical Methods

Organic transformations were carried out at ambient temperature unless indicated otherwise. Organic solvents (Sigma-Aldrich, Merck Group, Darmstadt, Germany) used in this study were of reagent grade and were used without purification. All other commercially available reagents were of the highest purity (Sigma-Aldrich). All workup and purification procedures were carried out with reagent-grade solvents under ambient atmosphere.

Mass spectra were recorded on a UPLC-MS/MS system consisted of a Waters ACQUITY^®^ UPLC^®^ (Waters Corporation, Milford, MA, USA) coupled to a Waters TQD mass spectrometer (electrospray ionization mode ESI-tandem quadrupole). Chromatographic separations were carried out using the Acquity UPLC BEH (bridged ethyl hybrid) C18 column; 2.1 × 100 mm, and 1.7 µm particle size, equipped with Acquity UPLC BEH C18 VanGuard precolumn (Waters Corporation, Milford, MA, USA); 2.1 × 5 mm, and 1.7 µm particle size. The column was maintained at 40 °C, and eluted under gradient conditions from 95% to 0% of eluent A over 10 min, at a flow rate of 0.3 mL∙min^−1^. Eluent A: water/formic acid (0.1%, *v*/*v*); eluent B: acetonitrile/formic acid (0.1%, *v*/*v*). Chromatograms were made using Waters eλ PDA detector. Spectra were analyzed in the 200–700 nm range with 1.2 nm resolution and sampling rate 20 points/s. MS detection settings of Waters TQD mass spectrometer were as follows: source temperature 150 °C, desolvation temperature 350 °C, desolvation gas flow rate 600 L∙h^−1^, cone gas flow 100 L∙h^−1^, capillary potential 3.00 kV, cone potential 40 V. Nitrogen was used for both nebulizing and drying gas. The data were obtained in a scan mode ranging from 50 to 1000 *m*/*z* in time 0.5 s intervals. Data acquisition software was MassLynx V 4.1 (Waters Corporation, Milford, MA, USA). The UPLC/MS purity of all the final compounds was confirmed to be 95% or higher.

^1^H-NMR and ^13^C-NMR spectra were obtained in Varian BB 300 spectrometer (Varian, Palo Alto, CA, USA) in CDCl_3_ or *d*_6_-DMSO, and were recorded at 300 and 75 MHz, respectively. The *J* values are reported in Hertz (Hz), and the splitting patterns are designated as follows: s (singlet), br.s. (broad singlet), d (doublet), t (triplet), q (quartet), dd (doublet of doublets), dt (doublet of triplets), td (triplet of doublets), ddd (doublet of doublet of doublets), m (multiplet).

Elemental analyses for C, H, N and S were carried out using the elemental Vario EL III Elemental Analyser (Elementar Analysensysteme GmbH, Hanau, Germany). All values are given as percentages, and were within ±0.4% of the calculated values.

Melting points (mp) were determined with a Büchi apparatus (Flawil, Switzerland) and are uncorrected.

The general procedures used for the synthesis of intermediate and final compounds were in accordance with previously reported methodology [20]. 

Spectroscopic data (MS, ^1^H-NMR and ^13^C-NMR spectra) for representative final compounds are presented in Supplementary Materials.

#### 7.1.2. Preparation of 1-Methoxy-2-(2,2,2-trifluoroethoxy)benzene (**2**)

2-Methoxy-phenol **1** (5.19 g, 0.04 mol) was dissolved in DMF (25 mL), after addition of K_2_CO_3_, (16.6 g, 0.12 mol) a mixture that was heated to 90 °C. Then 2-iodo-1,1,1-trifluoroethane (4.2 mL, 0.05 mol) was added dropwise in 30 min. The reaction mixture was then heated under reflux for 24 h. Inorganic residues were filtered off and organic mixture was concentrated under reduced pressure. The obtained crude product was purified using silica gel with AcOEt/Hexane (1/9, *v*/*v*) as an eluting system (isolated yield 65%). Yellow oil (5.6 g); UPLC/MS purity 99%, *t*_R_ = 6.52. C_9_H_9_F_3_O_2_, MW 206.16, Monoisotopic Mass 206.06, [M + H]^+^ 207.1. ^1^H-NMR (300 MHz, CDCl_3_) δ 3.91 (s, 3H, O–CH_3_), 4.36 (q, *J* = 8.4 Hz, 2H, O–CH_2_–CF_3_), 6.81–7.11 (m, 4H, Ar–H). ^13^C-NMR (75 MHz, CDCl_3_) δ 56.4, 68.3, 119.0, 121.5, 122.3, 123.8, 126.1, 127.2, 138.9.

#### 7.1.3. Preparation of 2-(2,2,2-Trifluoroethoxy)phenol (**3**)

A 1 M solution of boron tribromide (30 mL, 0.03 mol) in CH_2_Cl_2_ was added to a solution of intermediate **2** (4.3 g, 0.02 mol) in anhydrous CH_2_Cl_2_ (50 mL) at −20 °C. The reaction mixture was warmed to room temperature and, after, quenched by addition of excess saturated aqueous sodium bicarbonate solution (40 mL). The organic layer was dried over Na_2_SO_4_, filtered, and concentrated under reduced pressure. The crude material was sufficiently pure to be used directly in the next step (yield 98%). Yellow oil (3.8 g); UPLC/MS purity 99%, t_R_ = 5.37. C_8_H_7_F_3_O_2_, MW 192.14, Monoisotopic Mass 192.04, [M + H]^−^ 191.0. ^1^H-NMR (300 MHz, CDCl_3_) δ 4.36 (q, *J* = 8.4 Hz, 2H, O–CH_2_–CF_3_), 6.81–7.11 (m, 4H, Ar–H), 9.72 (br.s., 1H, O–H). ^13^C-NMR (75 MHz, CDCl_3_) δ 56.2, 68.1, 119.2, 121.5, 122.4, 123.7, 126.4, 127.3, 136.4

#### 7.1.4. Preparation of 1-(2-Bromoethoxy)-2-(2,2,2-trifluoroethoxy)benzene (**4**)

Phenol **3** (4.8 g, 0.025 mol) was dissolved in acetone (30 mL). Then K_2_CO_3_ (10.4 g, 0.075 mol) and catalytic amount of KI (0.08 g, 0.0005 mol) were added, followed by dropwise addition of 1,2-dibromoethane (12.9 mL, 0.15 mol). The reaction was refluxed for 48 h. Inorganic residues were filtered off and organic mixture was concentrated under reduced pressure. The obtained crude product was purified using silica gel with AcOEt/Hexane (0.5/9, *v*/*v*) as an eluting system (isolated yield 75%). Yellow oil (5.61 g); UPLC/MS purity 97%, *t*_R_ = 7.41. C_10_H_10_BrF_3_O_2_, MW 299.09, Monoisotopic Mass 297.98, [M + H]^+^ 300.2. ^1^H-NMR (300 MHz, CDCl_3_) δ 3.45 (t, *J* = 6.5 Hz, 2H, N–CH_2_–CH_2_), 4.29 (t, *J* = 6.5 Hz, 2H, O–CH_2_–CH_2_), 4.36 (q, *J* = 8.4 Hz, 2H, O–CH_2_–CF_3_), 6.82 (dd, *J* = 5.2, 1.0 Hz, 1H, Ar–H), 6.99 (td, *J* = 7.6, 1.1 Hz, 1H, Ar–H), 7.14 (td, *J* = 8.0, 1.8 Hz, 1H, Ar–H), 7.24–7.28 (m, 1H, Ar–H). ^13^C-NMR (75 MHz, CDCl_3_) δ 56.2, 64.1, 65.8, 68.1, 119.3, 121.5, 122.4, 123.7, 126.4, 127.3, 136.4.

#### 7.1.5. General Procedure for the Alkylation of Boc-Protected Amines (**5**–**7**)

Commercial Boc-protected amines (1 eq) were dissolved in acetone (15 mL). Then, K_2_CO_3_ (3 eq) and a catalytic amount of KI (0.02 eq) were added, followed by dropwise addition of (aryloxy)ethyl bromide **4** (1.2 eq) in 30 min. The reaction was heated under reflux for 48 h. Inorganic residues were filtered off and organic mixture was concentrated under reduced pressure. The obtained crude products were purified according to the methods described below (isolated yields 68–75%).

##### *tert*-Butyl ((1-(2-(2-(2,2,2-trifluoroethoxy)phenoxy)ethyl)piperidin-4-yl)methyl)carbamate (**5**)

Compound **5** was prepared using 4-Boc-aminomethyl-piperidine (1.1 g, 5.8 mmol), K_2_CO_3_ (2.4 g, 17.4 mmol), KI (0.02 g, 0.12 mmol) and (aryloxy)ethyl bromide **4** (2.1 g, 6.96 mmol). Yellow oil 2.06 g, (isolated yield 68%), following chromatographic purification over silica gel with CH_2_Cl_2_/MeOH (9/0.7, *v*/*v*); UPLC/MS purity 97%, *t*_R_ = 4.57. C_21_H_31_F_3_N_2_O_4_, MW 432.48, Monoisotopic Mass 432.22, [M + H]^+^ 433.5. ^1^H-NMR (300 MHz, CDCl_3_) δ 1.08–1.20 (m, 2H), 1.34–1.42 (m, 1H, piperidine), 1.45 (s, 9H, (CH_3_)_3_–C), 1.56–1.60 (m, 2H, piperidine), 1.92–1.99 (m, 2H, piperidine), 2.79–2.88 (m, 4H, piperidine), 3.45 (t, *J* = 6.5 Hz, 2H, N–CH_2_–CH_2_), 4.29 (t, *J* = 6.5 Hz, 2H, O–CH_2_–CH_2_), 4.36 (q, *J* = 8.4 Hz, 2H, O–CH_2_–CF_3_), 4.52 (br.s, 1H, SO_2_–NH–CH_2_), 6.82 (dd, *J* = 5.2, 1.0 Hz, 1H, Ar–H), 6.99 (td, *J* = 7.6, 1.1 Hz, 1H, Ar–H), 7.14 (td, *J* = 8.0, 1.8 Hz, 1H, Ar–H), 7.24–7.28 (m, 1H, Ar–H). ^13^C-NMR (75 MHz, CDCl_3_) δ 27.0, 28.3, 34.0, 47.6, 52.6, 55.4, 64.1, 65.8, 66.3, 79.2, 120.7, 122.1,122.7, 123.4, 126.3, 128.9, 135.6, 155.3.

##### *tert-*Butyl (*R*)-(1-{2-[2-(2,2,2-trifluoroethoxy)phenoxy]ethyl}pyrrolidin-3-yl)carbamate (**6**)

Compound **6** was prepared using (*R*)-3-Boc-amino-pyrrolidine (0.75 g, 4.03 mmol), K_2_CO_3_ (1.67 g, 12.09 mmol), KI (0.01 g, 0.08 mmol), and (aryloxy)ethyl bromide **4** (1.5 g, 4.84 mmol). Yellow oil 1.17 g, (isolated yield 72%), following chromatographic purification over silica gel with CH_2_Cl_2_/MeOH (9/0.7 *v*/*v*); UPLC/MS purity 97%, *t*_R_ = 4.72. C_19_H_27_F_3_N_2_O_4_, MW 404.43, Monoisotopic Mass 404.19, [M + H]^+^ 405.1. ^1^H-NMR (300 MHz, CDCl_3_) δ 1.43 (2, 9H, (CH_3_)_3_–C), 1.51–1.64 (m, 1H, pyrrolidine), 2.04–2.18 (m, 1H, piperidine), 2.33 (td, *J* = 8.91, 7.16 Hz, 1H, piperidine), 2.71–2.92 (m, 4H, piperidine), 3.45 (t, *J* = 6.5 Hz, 2H, N–CH_2_–CH_2_), 3.79 (br.s, 1H, SO_2_–NH–CH), 4.29 (t, *J* = 6.5 Hz, 2H, O–CH_2_–CH_2_), 4.36 (q, *J* = 8.4 Hz, 2H, O–CH_2_–CF_3_), 6.82 (dd, *J* = 5.2, 1.0 Hz, 1H, Ar–H), 6.99 (td, *J* = 7.6, 1.1 Hz, 1H, Ar–H), 7.14 (td, *J* = 8.0, 1.8 Hz, 1H, Ar–H), 7.24–7.28 (m, 1H, Ar–H). ^13^C-NMR (75 MHz, CDCl_3_) δ 28.3, 29.0, 32.5, 52.7, 54.0, 60.8, 64.1, 65.8, 66.3, 79.2, 120.7, 122.1,122.7, 123.4, 126.3, 128.9, 135.6, 156.1.

##### *tert-*Butyl (*S*)-(1-(2-(2-(2,2,2-trifluoroethoxy)phenoxy)ethyl)pyrrolidin-3-yl)carbamate (**7**)

Compound **7** was prepared using (*S*)-3-Boc-amino-pyrrolidine (0.75 g, 4.03 mmol), K_2_CO_3_ (1.67 g, 12.09 mmol), KI (0.01 g, 0.08 mmol), and (aryloxy)ethyl bromide **4** (1.5 g, 4.84 mmol). Yellow oil 1.22 g, (isolated yield 75%), following chromatographic purification over silica gel with CH_2_Cl_2_/MeOH (9/0.7 *v*/*v*); UPLC/MS purity 98%, *t*_R_ = 4.84. C_19_H_27_F_3_N_2_O_4_, MW 404.43, Monoisotopic Mass 404.19, [M + H]^+^ 405.3. ^1^H-NMR (300 MHz, CDCl_3_) δ 1.43 (2, 9H, (CH_3_)_3_–C), 1.51–1.64 (m, 1H, pyrrolidine), 2.04–2.18 (m, 1H, pyrrolidine), 2.33 (td, *J* = 8.91, 7.16 Hz, 1H, pyrrolidine), 2.71–2.92 (m, 4H, pyrrolidine), 3.45 (t, *J* = 6.5 Hz, 2H, N–CH_2_–CH_2_), 3.79 (br.s, 1H, SO_2_–NH–CH), 4.29 (t, *J* = 6.5 Hz, 2H, O–CH_2_–CH_2_), 4.36 (q, *J* = 8.4 Hz, 2H, O–CH_2_–CF_3_), 6.82 (dd, *J* = 5.2, 1.0 Hz, 1H, Ar–H), 6.99 (td, *J* = 7.6, 1.1 Hz, 1H, Ar–H), 7.14 (td, *J* = 8.0, 1.8 Hz, 1H, Ar–H), 7.24–7.28 (m, 1H, Ar–H). ^13^C-NMR (75 MHz, CDCl_3_) δ 28.2, 29.0, 32.4, 52.7, 54.0, 60.7, 64.1, 65.8, 66.1, 79.2, 120.7, 122.0,122.7, 123.5, 126.3, 128.9, 135.3, 156.5.

#### 7.1.6. General Procedure for Preparation of Final Compounds (**8**–**18**)

Intermediates **5**–**7** were converted into their TFA salts by treatment with a mixture of TFA/CH_2_Cl_2_ (4 mL/1 mL). The excess reagent and solvent were removed under reduced pressure and maintained overnight under vacuum. A mixture of the appropriate (aryloxy)ethyl alicyclic amine (1 eq) in CH_2_Cl_2_ (3 mL) and TEA (3 eq) was then cooled in an ice bath, and the proper arylsulfonyl chloride (1.2 eq) was added at 0 °C (the entire amount was added at the same time). The reaction mixture was stirred for 2–6 h under cooling. The solvent was evaporated, and the sulfonamides were a purified silica-gel column with CH_2_Cl_2_/MeOH (9/0.7, *v*/*v*) as an eluting system (isolated yields 55–87%). Compounds **9** and **10**, selected for *in vivo* pharmacological evaluation, were further converted into the hydrochloride salts by treatment of their solution in anhydrous ethanol with 1.25 M HCl in MeOH.

##### 4-Fluoro-*N*-[(1-{2-[(2,2,2-trifluoroethoxy)phenoxy]ethyl}piperidin-4-yl)methyl]benzenesulfonamide (**8**)

Compound **8** was prepared using intermediate **5** (150 mg, 0.45 mmol), TEA (0.19 mL, 1.35 mmol), and 4-fluorobenzenesulfonyl chloride (110 mg, 0.54 mmol). Yellow oil 200 mg (isolated yield 87%); UPLC/MS purity 100%, *t*_R_ = 4.92. C_22_H_26_F_4_N_2_O_4_S, MW 490.51, Monoisotopic Mass 490.15, [M + H]^+^ 491.4. ^1^H-NMR (300 MHz, CDCl_3_) δ 1.25–1.37 (m, 2H, piperidine), 1.51–1.54 (m, 1H, piperidine), 1.70 (d, *J* = 13.5 Hz, 2H, piperidine), 2.22 (t, *J* = 11.5 Hz, 2H, piperidine), 2.83 (t, *J* = 6.5 Hz, 2H, piperidine), 2.91 (t, *J* = 5.4 Hz, 2H, N–CH_2_–CH_2_), 3.12 (d, *J* = 11.3 Hz, 2H, NH–CH_2_–CH), 4.17 (t, *J* = 5.5 Hz, 2H, O–CH_2_–CH_2_), 4.38 (q, *J* = 8.4 Hz, 2H, O–CH_2_–CF_3_), 6.88–6.95 (m, 3H, Ar–H), 6.96–7.06 (m, 1H, Ar–H), 7.14–7.22 (m, 2H, Ar–H), 7.83–7.91 (m, 2H, Ar–H). Anal. calcd for C_22_H_26_F_4_N_2_O_4_S HCl: C: 50.14, H: 5.16, N: 5.32, S: 6.08; Found C: 49.96, H: 4.96, N: 5.17, S: 5.78. Mp for C_22_H_26_F_4_N_2_O_4_S HCl:155.5–158.2 °C.

##### 3-Chloro-2-fluoro-*N*-[(1-{2-[(2,2,2-trifluoroethoxy)phenoxy]ethyl}piperidin-4-yl)methyl]benzenesulfonamide (**9**)

Compound **9** was prepared using intermediate **5** (150 mg, 0.45 mmol), TEA (0.19 mL, 1.35 mmol), and 3-chloro-2-fluorobenzenesulfonyl chloride (0.08 mL, 0.54 mmol). Yellow oil, 190 mg (isolated yield 82%); UPLC/MS purity 100%, *t*_R_ = 5.35. C_22_H_25_ClF_4_N_2_O_4_S, MW 524.96, Monoisotopic Mass 524.12, [M + H]^+^ 525.3. ^1^H-NMR (300 MHz, CDCl_3_) δ 1.15–1.30 (m, 2H, piperidine), 1.48–1.55 (m, 1H, piperidine), 1.70 (d, *J* = 13.1 Hz, 2H, piperidine), 2.11 (td, *J* = 11.7, 2.0 Hz, 2H, piperidine), 2.82 (t, *J* = 5.7 Hz, 2H, piperidine), 2.88 (t, *J* = 6.2 Hz, 2H, N–CH_2_–CH_2_), 3.02 (d, *J* = 11.7 Hz, 2H, NH–CH_2_–CH), 4.12 (t, *J* = 5.7 Hz, 2H, O–CH_2_–CH_2_), 4.39 (q, *J* = 8.5 Hz, 2H, O–CH_2_–CF_3_), 6.89–6.93 (m, 2H, Ar–H), 6.96–7.06 (m, 2H, Ar–H), 7.19–7.25 (m, 1H, Ar–H), 7.62 (ddd, *J* = 8.2, 6.7, 1.7 Hz, 1H, Ar–H), 7.79 (ddd, *J* = 7.9, 6.3, 1.7 Hz, 1H, Ar–H). ^13^C-NMR (75 MHz, DMSO-*d*_6_) δ 27.0, 34.0, 47.6, 52.6, 55.4, 64.1, 65.8, 66.3, 115.0 (d, *J* = 80.6 Hz), 121.7, 122.1 (d, *J* = 10.4 Hz), 122.7, 123.4, 126.2 (d, *J* = 4.6 Hz), 126.3, 128.9, 130.5 (d, *J* = 13.8 Hz), 135.6, 147.1 (d, *J* = 58.7 Hz), 154.0 (d, *J* = 255.7 Hz). Anal. calcd for C_22_H_25_ClF_4_N_2_O_4_S HCl: C: 47.07, H: 4.67, N: 4.99, S: 5.71; Found C: 47.09, H: 4.48, N: 4.74, S: 5.32. Mp for C_22_H_25_ClF_4_N_2_O_4_S HCl: 163.1–164.9 °C.

##### 5-Chloro-2-fluoro-*N*-[(1-{2-[(2,2,2-trifluoroethoxy)phenoxy]ethyl}piperidin-4-yl)methyl]benzenesulfonamide (**10**)

Compound **10** was prepared using intermediate **5** (150 mg, 0.45 mmol), TEA (0.19 mL, 1.35 mmol), and 5-chloro-2-fluorobenzenesulfonyl chloride (120 mg, 0.54 mmol). Yellow oil, 190 mg (isolated yield 81%); UPLC/MS purity 100%, *t*_R_ = 5.41. C_22_H_25_ClF_4_N_2_O_4_S, MW 524.96, Monoisotopic Mass 524.12, [M + H]^+^ 525.3. ^1^H-NMR (300 MHz, CDCl_3_) δ 1.17–1.32 (m, 2H, piperidine), 1.43–1.57 (m, 1H, piperidine), 1.70 (d, *J* = 13.3 Hz, 2H, piperidine), 2.08–2.18 (m, 2H, piperidine), 2.83 (t, *J* = 5.7 Hz, 2H, piperidine), 2.87 (t, *J* = 6.2 Hz, 2H, N–CH_2_–CH_2_), 3.04 (d, *J* = 11.8 Hz, 2H, NH–CH_2_–CH), 4.13 (t, *J* = 5.6 Hz, 2H, O–CH_2_–CH_2_), 4.41 (q, *J* = 8.5 Hz, 2H, O–CH_2_–CF_3_), 4.90 (br.s., 1H, SO_2_–NH–CH_2_), 6.89–6.93 (m, 2H, Ar–H), 6.96–7.06 (m, 2H, Ar–H), 7.12–7.19 (m, 1H, Ar–H), 7.52 (ddd, *J* = 8.8, 4.3, 2.7 Hz, 1H, Ar–H), 7.87 (dd, *J* = 6.1, 2.7 Hz, 1H, Ar–H). ^13^C-NMR (75 MHz, DMSO-*d*_6_) δ 27.0, 34.0, 47.5, 52.6, 55.4, 64.1, 65.6, 66.2, 115.0 (d, *J* = 74.9 Hz), 119.9 (d, *J* = 21.9 Hz), 122.1, 123.4, 129.1 (d, *J* = 3.5 Hz), 130.4 (d, *J* = 16.1 Hz), 135.3 (d, *J* = 9.2 Hz), 147.4 (d, *J* = 57.6 Hz), 157.3 (d, *J* = 249.9 Hz). Anal. calcd for C_22_H_25_ClF_4_N_2_O_4_S HCl: C: 47.07, H: 4.67, N: 4.99, S: 5.71; Found C: 47.17, H: 4.32, N: 4.74, S: 5.39. Mp for C_22_H_25_ClF_4_N_2_O_4_S HCl: 165.0–166.6 °C.

##### 5-Chloro-2-methoxy-*N*-[(1-{2-[(2,2,2-trifluoroethoxy)phenoxy]ethyl}piperidin-4-yl)methyl]benzenesulfonamide (**11**)

Compound **11** was prepared using intermediate **5** (150 mg, 0.45 mmol), TEA (0.19 mL, 1.35 mmol), and 5-chloro-2-methoxybenzenesulfonyl chloride (130 mg, 0.54 mmol). Yellow oil, 140 mg (isolated yield 58%); UPLC/MS purity 98%, *t*_R_ = 5.35. C_23_H_28_ClF_3_N_2_O_5_S, MW 536.14, Monoisotopic Mass 536.99, [M + H]^+^ 537.2. ^1^H-NMR (300 MHz, CDCl_3_) δ 1.14–1.29 (m, 2H, piperidine), 1.50–1.58 (m, 1H, piperidine), 1.70 (d, *J* = 12.5 Hz, 2H, piperidine), 2.17 (t, *J* = 11.0 Hz, 2H, piperidine), 2.73 (t, *J* = 6.6 Hz, 2H, piperidine), 2.86 (t, *J* = 5.5 Hz, 2H, N–CH_2_–CH_2_), 3.04–3.11 (m, 2H, NH–CH_2_–CH), 3.95 (s, 3H, O–CH_3_), 4.15 (t, *J* = 5.5 Hz, 2H, O–CH_2_–CH_2_), 4.38 (q, *J* = 8.4 Hz, 2H, O–CH_2_–CF_3_), 5.04 (br.s., 1H, SO_2_–NH–CH_2_), 6.94 (dd, *J* = 16.5, 8.1 Hz, 4H, Ar–H), 6.99–7.05 (m, 1H, Ar–H), 7.48 (dd, *J* = 8.9, 2.7 Hz, 1H, Ar–H), 7.87 (d, *J* = 2.7 Hz, 1H, Ar–H). Anal. calcd for C_23_H_28_ClF_3_N_2_O_5_S: C: 51.44, H: 5.26, N: 5.22, S: 5.97; Found C: 51.13, H: 5.06, N: 5.07, S: 5.65.

##### 3,4-Dimethoxy-*N*-[(1-{2-[(2,2,2-trifluoroethoxy)phenoxy]ethyl}piperidin-4-yl)methyl]benzenesulfonamide (**12**)

Compound **12** was prepared using intermediate **5** (150 mg, 0.45 mmol), TEA (0.19 mL, 1.35 mmol), and 3,4-dimethoxybenzenesulfonyl chloride (130 mg, 0.54 mmol). Yellow oil, 180 mg (isolated yield 77%); UPLC/MS purity 98%, *t*_R_ = 4.71. C_24_H_31_F_3_N_2_O_6_S, MW 532.57, Monoisotopic Mass 532.19, [M + H]^+^ 533.4. ^1^H-NMR (300 MHz, CDCl_3_) δ 1.14–1.29 (m, 2H, piperidine), 1.46–1.54 (m, 1H, piperidine), 1.67 (d, *J* = 12.8 Hz, 2H, piperidine), 2.11 (t, *J* = 11.0 Hz, 2H, piperidine), 2.77–2.80 (m, 2H,), 2.82–2.86 (m, 2H, N–CH_2_–CH_2_), 3.02 (d, *J* = 11.5 Hz, 2H, NH–CH_2_–CH), 3.91 (s, 3H, O–CH_3_), 3.93 (s, 3H, O–CH_3_), 4.12 (t, *J* = 5.6 Hz, 2H, O–CH_2_–CH_2_), 4.39 (q, *J* = 8.5 Hz, 2H, O–CH_2_–CF_3_), 4.68 (br.s., 1H, SO_2_–NH–CH_2_), 6.86–6.97 (m, 4H, Ar–H), 6.98–7.05 (m, 1H, Ar–H), 7.33 (d, *J* = 2.1 Hz, 1H, Ar–H), 7.46 (dd, *J* = 8.5, 2.2 Hz, 1H, Ar–H). Anal. calcd for C_24_H_31_F_3_N_2_O_6_S: C: 54.13, H: 5.87, N: 5.26, S: 6.02; Found C: 54.33, H: 6.01, N: 5.45, S: 6.34.

##### (*R*)-4-Fluoro-*N*-(1-{2-[(2,2,2-trifluoroethoxy)phenoxy]ethyl}pyrrolidin-3-yl)benzenesulfonamide (**13**)

Compound **13** was prepared using intermediate **6** (150 mg, 0.5 mmol), TEA (0.21 mL, 1.5 mmol), and 4-fluorobenzenesulfonyl chloride (120 mg, 0.6 mmol). Yellow oil, 150 mg (isolated yield 65%); UPLC/MS purity 95%, *t*_R_ = 5.07. C_20_H_22_F_4_N_2_O_4_S, MW 462.46, Monoisotopic Mass 462.12, [M + H]^+^ 463.3. ^1^H-NMR (300 MHz, CDCl_3_) δ 1.52–1.64 (m, 2H, pyrrolidine), 2.09–2.11 (m 1H, pyrrolidine), 2.31–2.41 (m, 1H, pyrrolidine), 2.58–2.64 (m, 1H, pyrrolidine), 2.82–2.86 (m, 2H, pyrrolidine), 2.93 (td, *J* = 9.0, 4.3 Hz, 2H, NH–CH_2_–CH_2_), 4.06 (t, *J* = 1.0 Hz, 2H, O–CH_2_–CH_2_), 4.36 (q, *J* = 8.4 Hz, 2H, O–CH_2_–CF_3_), 5.11 (br.s., 1H, SO_2_–NH–CH), 6.87–6.96 (m, 3H, Ar–H), 6.97–7.07 (m, 1H, Ar–H), 7.09–7.17 (m, 2H, Ar–H), 7.81–7.89 (m, 2H, Ar–H). ^13^C-NMR (75 MHz, CDCl_3_) δ 29.1, 32.5, 52.7, 54.0, 60.8, 67.7, 115.3 (d, *J* = 131.3 Hz), 116.8 (d, *J* = 59.9 Hz), 121.6, 121.7, 124.1, 129.7 (d, *J* = 9.2 Hz), 148.3 (d, *J* = 155.5 Hz), 165.0 (d, *J* = 252.2 Hz). Anal. calcd for C_20_H_22_F_4_N_2_O_4_S: C: 51.94, H: 4.80, N: 6.06, S: 6.93; Found C: 51.75, H: 4.64, N: 6.35, S: 6.97.

##### (*S*)-4-Fluoro-*N*-(1-{2-[(2,2,2-trifluoroethoxy)phenoxy]ethyl}pyrrolidin-3-yl)benzenesulfonamide (**14**)

Compound **14** was prepared using intermediate **7** (150 mg, 0.5 mmol), TEA (0.21 mL, 1.5 mmol), and 4-fluorobenzenesulfonyl chloride (120 mg, 0.6 mmol). Yellow oil, 140 mg (isolated yield 61%); UPLC/MS purity 95%, *t*_R_ = 4.80. C_20_H_22_F_4_N_2_O_4_S, MW 462.46, Monoisotopic Mass 462.12, [M + H]^+^ 463.2. ^1^H-NMR (300 MHz, CDCl_3_) δ 1.52–1.64 (m, 2H, pyrrolidine), 2.09–2.11 (m 1H, pyrrolidine), 2.31–2.41 (m, 1H, pyrrolidine), 2.58–2.64 (m, 1H, pyrrolidine), 2.82–2.86 (m, 2H, pyrrolidine), 2.93 (td, *J* = 9.0, 4.3 Hz, 2H, NH–CH_2_–CH_2_), 4.06 (t, *J* = 1.0 Hz, 2H, O–CH_2_–CH_2_), 4.36 (q, *J* = 8.4 Hz, 2H, O–CH_2_–CF_3_), 5.11 (br.s., 1H, SO_2_–NH–CH), 6.87–6.96 (m, 3H, Ar–H), 6.97–7.07 (m, 1H, Ar–H), 7.09–7.17 (m, 2H, Ar–H), 7.81–7.89 (m, 2H, Ar–H). Anal. calcd for C_20_H_22_F_4_N_2_O_4_S: C: 51.94, H: 4.80, N: 6.06, S: 6.93; Found C: 51.73, H: 4.62, N: 6.33, S: 6.95.

##### (*R*)-5-Chloro-2-fluoro-*N*-(1-{2-[(2,2,2-trifluoroethoxy)phenoxy]ethyl}pyrrolidin-3-yl)benzenesulfonamide (**15**)

Compound **15** was prepared using intermediate **6** (150 mg, 0.5 mmol), TEA (0.21 mL, 1.5 mmol), and 5-chloro-2-fluorobenzenesulfonyl chloride (140 mg, 0.6 mmol). Yellow oil, 170 mg (isolated yield 68%); UPLC/MS purity 95%, *t*_R_ = 5.19. C_20_H_21_ClF_4_N_2_O_4_S, MW 496.90, Monoisotopic Mass 496.08, [M + H]^+^ 497.3. ^1^H-NMR (300 MHz, CDCl_3_) δ 1.60–1.72 (m, 2H, pyrrolidine), 2.11–2.16 (m, 1H, pyrrolidine), 2.32–2.42 (m, 1H, pyrrolidine), 2.50–2.57 (m, 1H, pyrrolidine), 2.67–2.73 (m, 1H, pyrrolidine), 2.88 (dt, *J* = 8.0, 5.4 Hz, 2H, NH–CH_2_–CH_2_), 2.94–3.02 (m, 1H, pyrrolidine), 4.08 (t, *J* = 5.4 Hz, 2H, O–CH_2_–CH_2_), 4.34 (q, *J* = 8.3 Hz, 2H, O–CH_2_–CF_3_), 6.88–6.98 (m, 3H, Ar–H), 7.00–7.07 (m, 2H, Ar–H), 7.43 (ddd, *J* = 8.8, 4.3, 2.7 Hz, 1H, Ar–H), 7.87 (dd, *J* = 6.1, 2.7 Hz, 1H, Ar–H). Anal. calcd for C_20_H_21_ClF_4_N_2_O_4_S: C: 48.34, H: 4.26, N: 5.64, S: 6.45; Found C: 48.47, H: 4.55, N: 5.99, S: 6.75.

##### (*S*)-5-Chloro-2-fluoro-*N*-(1-{2-[(2,2,2-trifluoroethoxy)phenoxy]ethyl}pyrrolidin-3-yl)benzenesulfonamide (**16**)

Compound **16** was prepared using intermediate **7** (150 mg, 0.5 mmol), TEA (0.21 mL, 1.5 mmol), and 5-chloro-2-fluorobenzenesulfonyl chloride (140 mg, 0.6 mmol). Yellow oil, 130 mg (isolated yield 55%); UPLC/MS purity 98%, *t*_R_ = 5.20. C_20_H_21_ClF_4_N_2_O_4_S, MW 496.90, Monoisotopic Mass 496.08, [M + H]^+^ 497.2. ^1^H-NMR (300 MHz, CDCl_3_) δ 1.60–1.72 (m, 2H, pyrrolidine), 2.07–2.20 (m, 1H, pyrrolidine), 2.31–2.41 (m, 1H, pyrrolidine), 2.51–2.57 (m, 1H, pyrrolidine), 2.65–2.70 (m, 1H, pyrrolidine), 2.79–2.90 (m, 2H, NH–CH_2_–CH_2_), 2.92–3.00 (m, 1H, pyrrolidine), 3.95 (br. s., 1H, SO_2_–NH–CH), 4.07 (t, *J* = 5.2 Hz, 2H, O–CH_2_–CH_2_), 4.34 (q, *J* = 8.2 Hz, 2H, O–CH_2_–CF_3_), 6.87–6.97 (m, 3H, Ar–H), 7.03 (t, *J* = 8.7 Hz, 2H, Ar–H), 7.40–7.46 (m, 1H, Ar–H), 7.86 (dd, *J* = 5.7, 2.2 Hz, 1H, Ar–H). Anal. calcd for C_20_H_21_ClF_4_N_2_O_4_S: C: 48.34, H: 4.26, N: 5.64, S: 6.45; Found C: 48.49, H: 4.57, N: 6.02, S: 6.78.

##### (*R*)-3,4-Dimethoxy-*N*-(1-{2-[(2,2,2-trifluoroethoxy)phenoxy]ethyl}pyrrolidin-3-yl)benzenesulfonamide (**17**)

Compound **17** was prepared using intermediate **6** (150 mg, 0.5 mmol), TEA (0.21 mL, 1.5 mmol), and 3,4-dimethoxybenzenesulfonyl chloride (140 mg, 0.6 mmol). Yellow oil, 180 mg (isolated yield 74%); UPLC/MS purity 95%, *t*_R_ = 4.54. C_22_H_27_F_3_N_2_O_6_S, MW 504.52, Monoisotopic Mass 504.15, [M + H]^+^ 505.3. ^1^H-NMR (300 MHz, CDCl_3_) δ 1.52–1.64 (m, 2H, pyrrolidine), 2.04–2.17 (m, 1H, pyrrolidine), 2.33–2.42 (m, 1H, pyrrolidine), 2.52–2.59 (m, 1H, pyrrolidine), 2.60–2.65 (m, 1H, pyrrolidine), 2.86 (td, *J* = 5.5, 1.5 Hz, 2H, NH–CH_2_–CH_2_), 2.90–2.97 (m, 1H, pyrrolidine), 3.90 (s, 3H, O–CH_3_), 3.92 (s, 3H, O–CH_3_), 4.07 (t, *J* = 5.5 Hz, 2H, O–CH_2_–CH_2_), 4.36 (q, *J* = 8.4 Hz, 2H, O–CH_2_–CF_3_), 4.67 (br.s., 1H, SO_2_–NH–CH), 6.87–6.93 (m, 3H, Ar–H), 6.95 (dd, *J* = 3.3, 1.8 Hz, 1H, Ar–H), 7.00–7.06 (m, 1H, Ar–H), 7.31 (d, *J* = 2.2 Hz, 1H, Ar–H), 7.46 (dd, *J* = 8.5, 2.2 Hz, 1H, Ar–H). Anal. calcd for C_22_H_27_F_3_N_2_O_6_S: C: 52.37, H: 5.39, N: 5.55, S: 6.35; Found C: 52.19, H: 5.15, N: 5.24, S: 6.05

##### (*S*)-3,4-Dimethoxy-*N*-(1-{2-[(2,2,2-trifluoroethoxy)phenoxy]ethyl}pyrrolidin-3-yl)benzenesulfonamide (**18**)

Compound **18** was prepared using intermediate **7** (150 mg, 0.5 mmol), TEA (0.21 mL, 1.5 mmol), and 3,4-dimethoxybenzenesulfonyl chloride (140 mg, 0.6 mmol). Yellow oil, 170 mg (isolated yield 68%); UPLC/MS purity 97%, *t*_R_ = 4.57. C_22_H_27_F_3_N_2_O_6_S, MW 504.52, Monoisotopic Mass 504.15, [M + H]^+^ 505.3. ^1^H-NMR (300 MHz, CDCl_3_) δ 1.50–1.62 (m, 2H, pyrrolidine), 2.01–2.14 (m, 1H, pyrrolidine), 2.37 (td, *J* = 8.9, 6.9 Hz, 1H, pyrrolidine), 2.57 (d, *J* = 5.1 Hz, 2H, pyrrolidine), 2.80–2.85 (m, 2H, NH–CH_2_–CH_2_), 2.85–2.93 (m, 1H, pyrrolidine), 3.88 (s, 3H, O–CH_3_), 3.90 (s, 3H, O–CH_3_), 4.04 (t, *J* = 5.6 Hz, 2H, O–CH_2_–CH_2_), 4.35 (q, *J* = 8.4 Hz, 2H, O–CH_2_–CF_3_), 5.13 (br.s., 1H, SO_2_–NH–CH), 6.86–6.90 (m, 3H, Ar–H), 6.93 (dd, *J* = 5.6, 1.7 Hz, 1H, Ar–H), 6.96–7.05 (m, 1H, Ar–H), 7.32 (d, *J* = 2.2 Hz, 1H, Ar–H), 7.46 (dd, *J* = 8.5, 2.2 Hz, 1H, Ar–H). ^13^C-NMR (75 MHz, CDCl_3_) δ 32.4, 52.6, 52.7, 54.1, 56.1, 56.2, 60.8, 67.5, 67.7, 67.9, 109.5, 110.5, 114.4, 117.4, 120.9, 121.5, 124.0, 132.4, 147.3, 149.0, 152.4. Anal. calcd for C_22_H_27_F_3_N_2_O_6_S: C: 52.37, H: 5.39, N: 5.55, S: 6.35; Found C: 52.21, H: 5.18, N: 5.29, S: 6.09.

### 7.2. In Vitro Pharmacology

#### 7.2.1. Determination of the Affinity of the Tested Compounds at the α_1_- and α_2_-ARs

The affinity of the obtained compounds was evaluated by radioligand-binding assays (the ability to displace [^3^H]-Prazosin and [^3^H]-Clonidine from α_1_- and α_2_-ARs, respectively) on rat cerebral cortex. The brains are homogenized in 20 volumes of an ice-cold 50 mM Tris-HCl buffer (pH 7.6) and is centrifuged (MPW Med. Instruments, Warsaw, Poland) at 20,000 *g* for 20 min (0–4 °C). The cell pellet is resuspended in the Tris-HCl buffer and centrifuged again. Radioligand-binding assays are performed in plates (MultiScreen/Millipore, Burlington, MA, USA). The final incubation mixture (final volume 300 µL) consisted of 240 µL of the membrane suspension, 30 µL of [^3^H]-Prazosin (0.2 nM) or [^3^H]-Clonidine (2 nM) solution, and 30 µL of the buffer containing seven to eight concentrations (10^−11^ to 10^−4^ M) of the tested compounds. For measuring the unspecific binding, phentolamine, 10 µM (in the case of [^3^H]-Prazosin) and clonidine, and 10 µM (in the case of [^3^H]-Clonidine), were applied. The incubation was terminated by rapid filtration over glass fiber filters (Whatman GF/C, Sigma-Aldrich) using a vacuum manifold (Millipore). The filters were then washed twice with the assay buffer and placed in scintillation vials with a liquid-scintillation cocktail. Radioactivity was measured in a WALLAC 1409 DSA liquid-scintillation counter (BioSurplus, San Diego, CA, USA). All the assays were made in duplicate.

#### 7.2.2. Determination of the Affinity of the Tested Compounds at the 5-HT_1A_ and 5-HT_7_Rs

Binding experiments were conducted in 96-well microplates in a total volume of 250 μL of appropriate buffers. The composition of the assay buffers was as follows: 50 mM Tris-HCl, 0.1 mM EDTA, 10 mM MgCl_2_. The reaction mix included 50 μL solution of test compound, 50 μL of radioligand, and 150 μL of diluted membranes. All assays were incubated for 1 h (5-HT_1A_Rs) or 2 h (5-HT_7_Rs) at 37 °C. Radioactivity was counted in MicroBeta2 scintillation counter (PerkinElmer, Waltham, MA, USA). Nonspecific binding is defined with 10 µM of 5-HT and 10 µM of methiothepine in 5-HT_1A_R and 5-HT_7_R binding experiments, respectively. Each compound was tested in screening assay at two final concentrations of 10 µM and 1 µM.

#### 7.2.3. Determination of the Intrinsic Activity of the α_1A_-ARs

Intrinsic activity assay was performed according to the manufacturer of the assay kit (Invitrogen, Thermo Fisher Scientific corporation, Carlsbad, CA, USA). The cells were harvested and suspended in Assay Medium to a density of 312,500 cells/mL. Of the cell suspension, 32 µL per well was added to the Test Compound wells, the Unstimulated Control wells, and Stimulated Control wells and incubated per 16–24 h. To perform an agonist assay, 8 concentrations of 8 µL of the tested compound (10^−4^–10^−11^ M), e.g., in 5-fold higher concentration in comparison to the final tested concentration in the well, were added to the cells. To perform an antagonist assay, 8 concentrations of 4 µL of the tested compound (10^−4^–10^−11^M), e.g., in 10-fold higher concentration in comparison to the final tested concentration in the well, were added to the cells. Then, after 30 min, 4 µL of standard agonist in EC_80_ (10-fold higher concentration in comparison to the EC_80_ in the well), in Assay Medium, was added to the cells. Then, both the agonist and the antagonist plate were incubated in a humidified 37 °C/5% CO_2_ incubator for 5 h. After the incubation 8 µL of LiveBLAzer™-FRET B/G Substrate Mixture (CCF4-AM, Thermo Fisher Scientific corporation) was loaded cells in the absence of direct strong lighting, covered, and incubated at room temperature for 2 h.

#### 7.2.4. Determination of the Intrinsic Activity of the α_1B_-ARs and α_1D_-ARs

Intrinsic activity assay to α_1B_- and α_1D_-ARs was performed according to the manufacturer of the ready-to-use cells with stable expression of the α_1B_-adrenoreceptors and α_1D_-adrenoreceptors, respectively (PerkinElmer, Zaventem, Belgium). For measurement, cells (frozen, ready to use) were thawed and resuspended in 10 mL of assay buffer containing 5 µM coelenterazine h. This cells suspension was put in a 10 mL Falcon tube, fixed onto a rotating heel, and incubated for overnight at rt in the dark (8 rpm; 45° angle). Cells were diluted with Assay Buffer to 5000 cells/20 µL. Agonistic ligands 2 × (50 µL/well), diluted in Assay Buffer, were prepared in ½ white polystyrene area plates, and the cell suspension was dispensed in 50 µL volume on the ligands using the injector. The light emitted was recorded for 20 s. Cells with antagonist were incubated for 15 min at room temperature. Thereafter, 50 µL of agonist (3 × EC_80_ final concentration) was injected into the mix of cells and antagonist and the light emitted was recorded for 20 s.

#### 7.2.5. Determination of *In Vitro* Metabolic Stability

Metabolic stability of tested compound was analyzed using incubation systems, composed of: tested compounds (10 μM), RLMs (microsomes from rat male liver, pooled; 0.2 mg/mL; Sigma Aldrich), NADPH-regenerating system (NADP+, glucose-6-phosphate and glucose-6-phosphate dehydrogenase in 100mM potassium buffer, pH 7.4; all from Sigma Aldrich), and potassium buffer, pH 7.4. Stock solution of tested compounds was prepared in methanol (the final methanol concentration in incubation mixture does not exceed 0.2%). Firstly, all samples contained incubation mixture (without NADPH-regenerating system) were preincubated in a thermoblock at 37 °C for 10 min. Then reaction was initiated by the addition of NADPH-regenerating system. In control samples NADPH-regenerating system was replaced with potassium buffer. Probes were incubated in thermoblock for 15 and 30 min at 37 °C. After addition of internal standard (levallorphan, 10 μM), the biotransformation process was stopped by addition of perchloric acid (69–72%, by volume). Next, samples were centrifuged (Centrifuge 5427 R, Eppendorf, Hamburg, Germany) and supernatants were analyzed using UPLC/MS (Waters Corporation). All experiments were run in duplicates. Half-life time was evaluated using a nonlinear regression model using Graph Pad Prism software (Graph Pad Software, La Jolla, San Diego, CA, USA) and intrinsic clearance was calculated from the equation Cl_int_ = (volume of incubation [μL]/protein in the incubation [mg]) ×∙0.693/t_1/2_ [33]. 

### 7.3. In Vivo Pharmacology

#### 7.3.1. Animals

The experiments were carried out on male Wistar rats (body weight 200–250 g). The animals were housed in pairs in plastic cages in constant-temperature facilities exposed to a 12:12 h light/dark cycle; water and food were available ad libitum. Experimental groups consisted of 6 animals each. All experiments were conducted in accordance with the NIH Guide for Care and Use of Laboratory Animals and were approved by the Animal Use and Care Committee of the Jagiellonian University (2012, Kraków, Poland).

#### 7.3.2. Determination of the Effect of the Tested Compounds on Blood Pressure after a Single Administration in Rats

The normotensive rats were anesthetized with thiopental (70 mg/kg) by i.p. injection. The left carotid artery was cannulated with polyethylene tubing filled with heparin solution in saline to facilitate pressure measurements using PowerLab Apparatus (ADInstruments, Sydney, Australia). Blood pressure was measured: before administration of the compounds—time 0 min (control pressure), and 60 min thereafter. For each compound, studies were performed in the dose of 2 mg/kg b.w. Compounds were dissolved in water and administered intravenously. Initial blood pressure before administration of the tested compounds in all groups was similar.

#### 7.3.3. Statistical Analysis

Statistical calculations were carried out with the GraphPadPrism 6 program (GraphPad Software). Results are given as the arithmetic means with standard deviation (SD). The statistical significance was calculated using a one-way ANOVA and posthoc Bonfferoni Test in comparison to 0.9% NaCl. Differences were considered statistically significant at: * *p* ≤ 0.05, ** *p* ≤ 0.01, *** *p* ≤ 0.001.

## Figures and Tables

**Table 1:**
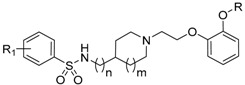
The biological data of compounds **8**–**18** for adrenergic α_1_- and α_2_-receptors.

Compd.	R_1_	Enant	n	m	R	*K*_i_ [nM] ^a^ ± SEM		
						α_1_	α_2_	S_α2/α1_^b^
**I** ^c^	3-Cl,2-F	-	1	1	Isopropyl	71 ± 4	1212 ± 99	17
**8**	4-F	-	1	1	TFE ^d^	20 ± 2	919 ± 25	46
**9**	3-Cl,2-F	-	1	1	TFE	50 ± 2	858 ± 69	17
**10**	5-Cl,2-F	-	1	1	TFE	26 ± 1	579 ± 20	23
**11**	5-Cl,2-OMe	-	1	1	TFE	95 ± 3	1092 ± 62	12
**12**	3,4-diOMe	-	1	1	TFE	19 ± 3	524 ± 31	27
**II** ^c^	5-Cl,2-F	S	0	0	Isopropyl	242 ± 16	>10,000	>41
**13**	4-F	R	0	0	TFE	188 ± 3	1687 ± 85	9
**14**	4-F	S	0	0	TFE	134 ± 16	>10,000	>70
**15**	5-Cl,2-F	R	0	0	TFE	171 ± 4	1188 ± 74	7
**16**	5-Cl,2-F	S	0	0	TFE	117 ± 10	1466 ± 80	13
**17**	3,4-diOMe	R	0	0	TFE	105 ± 7	435 ± 21	4
**18**	3,4-diOMe	S	0	0	TFE	70 ± 1	1141 ± 42	16

^a^*K*_i_ values based on two independent binding experiments; ^b^ Ratio of affinity for α_2_- and α_1_-ARs; ^c^ data taken from Reference [20]; ^d^ TFE = 2,2,2-trifluoroethyl.

**Table 2:** The functional activity of selected derivatives and reference drugs for α_1A_-, α_1B_-, and α_1D_-ARs.

Compd.	α_1A_		α_1B_		α_1D_	
	IC_50_ [nM] ^a^	Profile	IC_50_ [nM] ^a^	Profile	IC_50_ [nM] ^a^	Profile
**I** ^c^	11.1	ANT ^b^	42.1	ANT	15.1	ANT
**8**	3.8	ANT	8.3	ANT	1.1	ANT
**9**	0.8	ANT	3.9	ANT	1.1	ANT
**10**	2.1	ANT	10.1	ANT	2.7	ANT
**12**	15.0	ANT	0.02	ANT	2.6	ANT
**tamsulosin**	0.07	ANT	1.3	ANT	0.005	ANT
**terazosin**	51.9	ANT	1.7	ANT	0.2	ANT
**phenylephrine**	56.0 (EC_50_)	AGO ^b^	0.9 (EC_50_)	AGO	12.1 (EC_50_)	AGO

^a^ The EC_50_/IC_50_ values were based on three independent experiments; ^b^ ANT—antagonist, AGO—agonist.

**Table 3:** The binding data of selective compounds for 5-HT_1A_ and 5-HT_7_Rs.

Compd.	5-HT_1A_	5-HT_7_
	*K*_i_ [nM] ^a^ ± SEM	%inh @ 10^−6^/10^−7^ M
**9**	70 ± 3.5	93/57
**10**	46 ± 0.6	94/61

^a^*K*_i_ values based on two independent binding experiments.

**Table 4:** Metabolic stability of compounds **I**, **9**, **10** and reference drug tamsulosin.

Compd.	t_½_ [min]	Cl_int_ [μL/min/mg]	Major Metabolic Pathway
**I**	40	87	*N*-dealkylation
**9**	51.5	67	*N*-dealkylation
**10**	37.8	91.7	*N*-dealkylation
**Tamsulosin**	83	41	*N*-dealkylation

## Supplementary Materials

Supporting Information Available: MS, ^1^H-NMR and ^13^C-NMR spectra for representative final compounds.
