# Supplementary material for: Synthesis and Pharmacological Evaluation of Novel Silodosin-Based Arylsulfonamide Derivatives as α1A/α1D-Adrenergic Receptor Antagonist with Potential Uroselective Profile
Source: Molecules. 2018 Aug 29;23(9):2175. doi: 10.3390/molecules23092175 (PMC6225212; doi:10.3390/molecules23092175)

Supporting Information

Synthesis and pharmacological evaluation of novel silodosin-based arylsulfonamide derivatives as α_{1A}/α_{1D}-adrenergic receptor antagonist with potential uroselective profile

Vittorio Canale,[a] Aleksandra Rak,[b] Magdalena Kotańska,[b] Joanna Knutelska,[b] Agata Siwek,[c] Marek Bednarski,[b] Leszek Nowiński,[b] Małgorzata Zygmunt,[b] Paulina Koczurkiewicz,[d] Elżbieta Pękala,[d] Jacek Sapa,[b] Paweł Zajdel[a]

[a] Department of Medicinal Chemistry; [b] Department of Pharmacological Screening; [c] Department of Pharmacobiology; Faculty of Pharmacy, Jagiellonian University Medical College, 9 Medyczna Street, 30-688 Krakow, Poland

*Corresponding author, mail:
Jacek Sapa, jacek.sapa@uj.edu.pl
Department of Pharmacological Screening,
Faculty of Pharmacy, Jagiellonian University Medical College,
9 Medyczna Street, 30-688 Krakow, Poland

1. MS, ^{1}H NMR, and ^{13}C NMR spectra of representative compounds

1.1. *3-Chloro-2-fluoro-N-[(1-{2-[(2,2,2-trifluoroethoxy)phenoxy]ethyl}piperidin-4-yl)methyl]benzenesulfonamide* (**9**)

MS

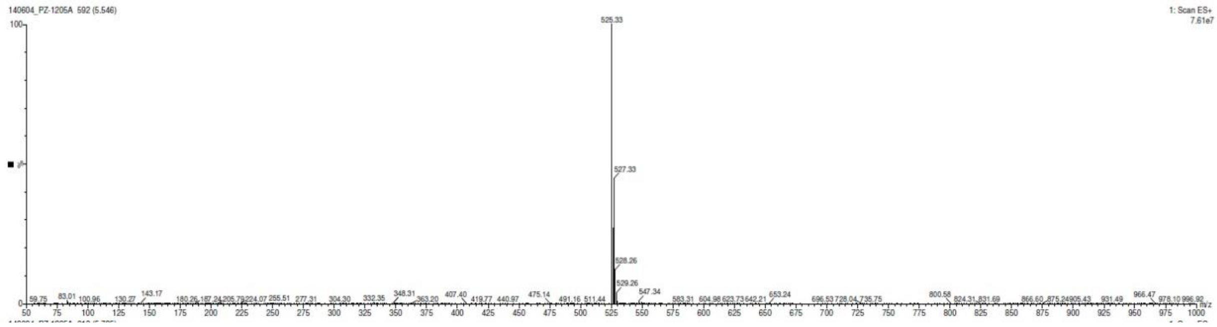

^{1}H-NMR 300 MHz, $CDCl_3$

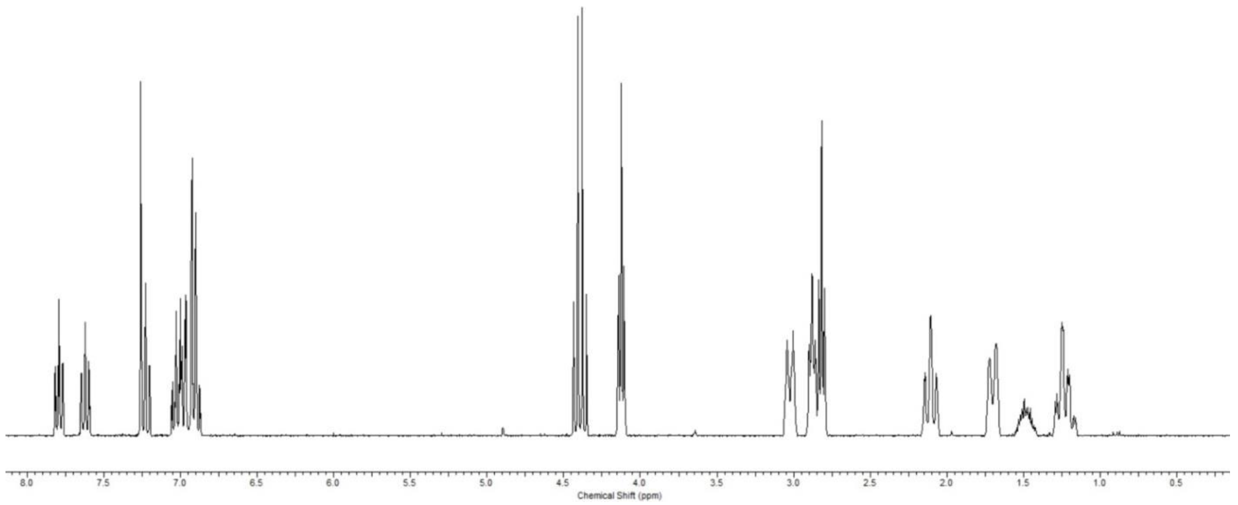

^{13}C-NMR 75 MHz, DMSO-d_6

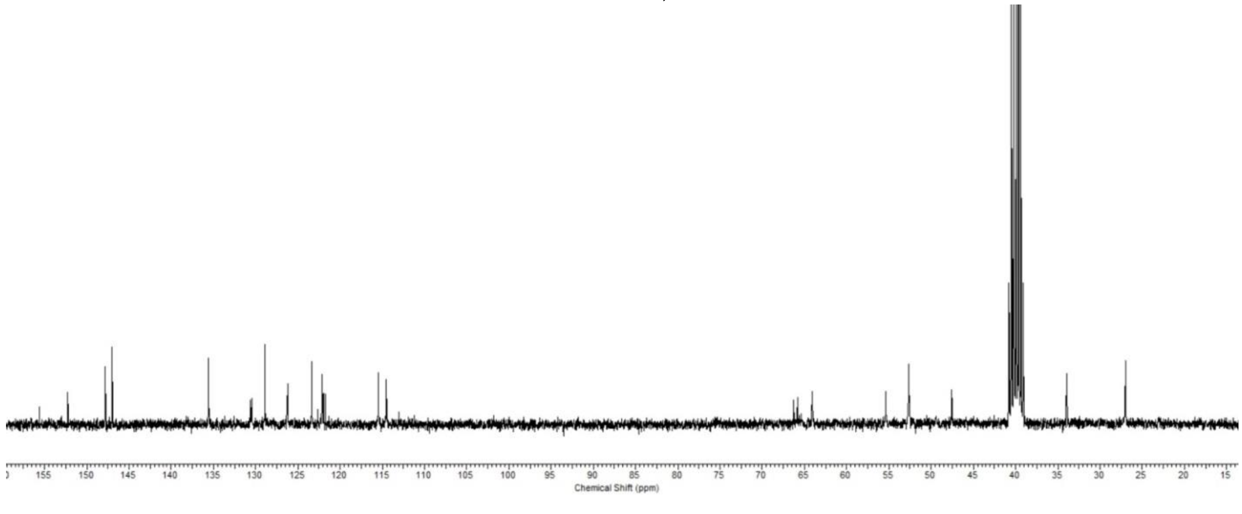

1.2. *5-Chloro-2-fluoro-N-[(1-{2-[(2,2,2-trifluoroethoxy)phenoxy]ethyl}piperidin-4-yl)methyl]benzenesulfonamide* (**10**)

MS

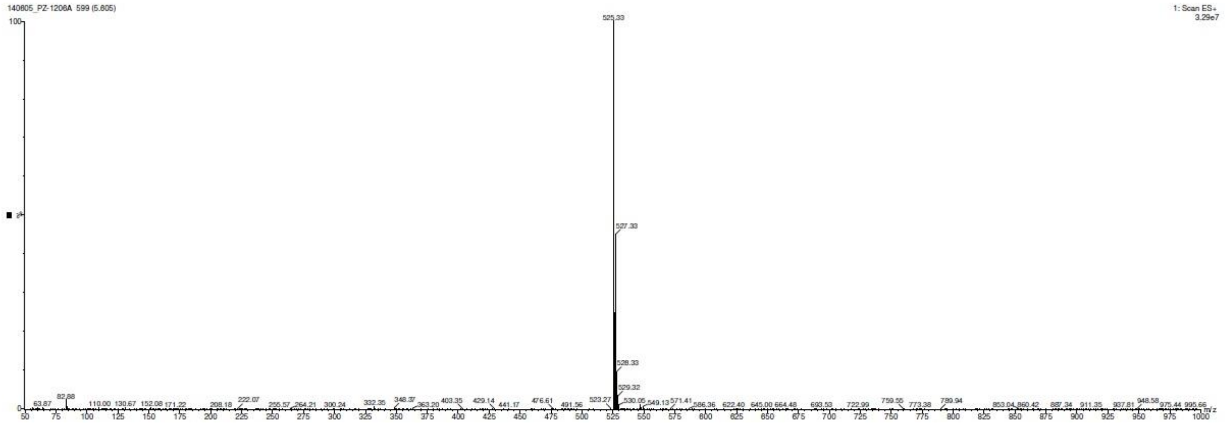

^{1}H-NMR 300 MHz, $CDCl_3$

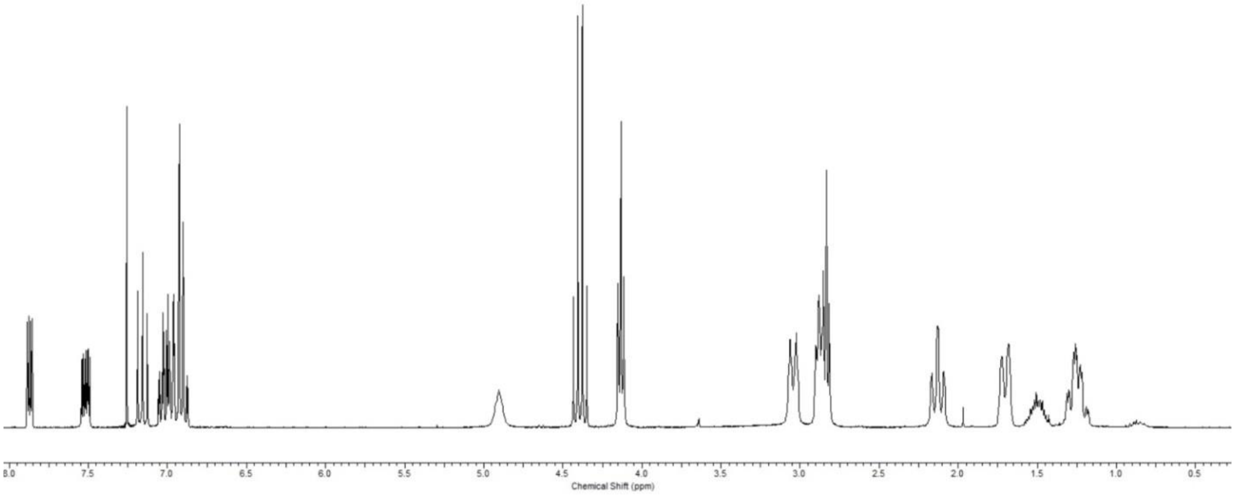

^{13}C-NMR 75 MHz, DMSO-d_6

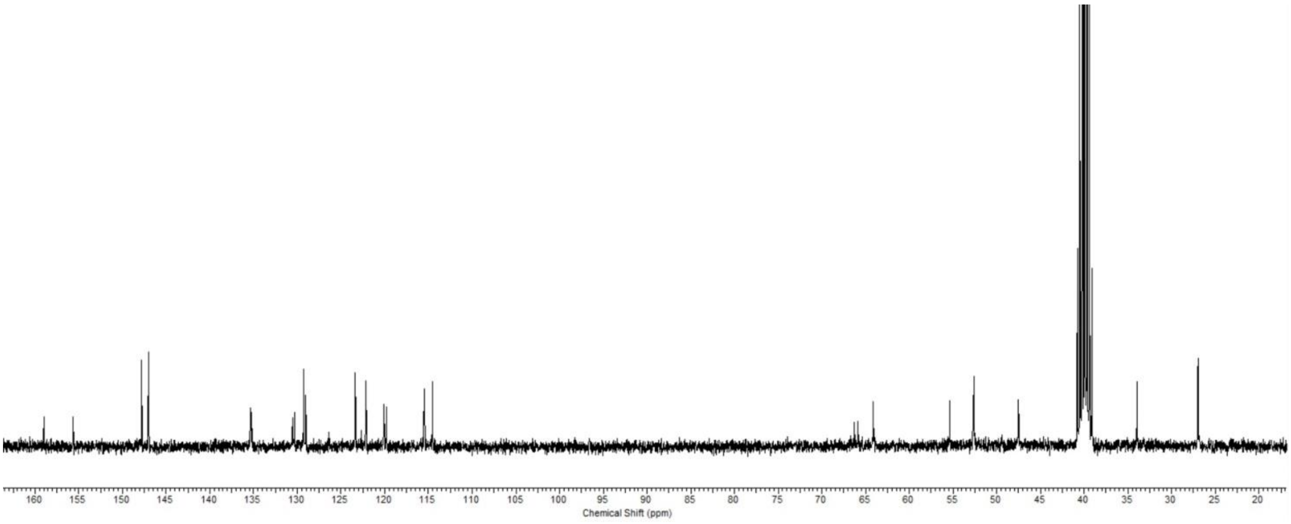

1.3. *(R)-4-Fluoro-N-(1-{2-[(2,2,2-trifluoroethoxy)phenoxy]ethyl}pyrrolidin-3-yl)benzenesulfonamide* (**13**)

MS

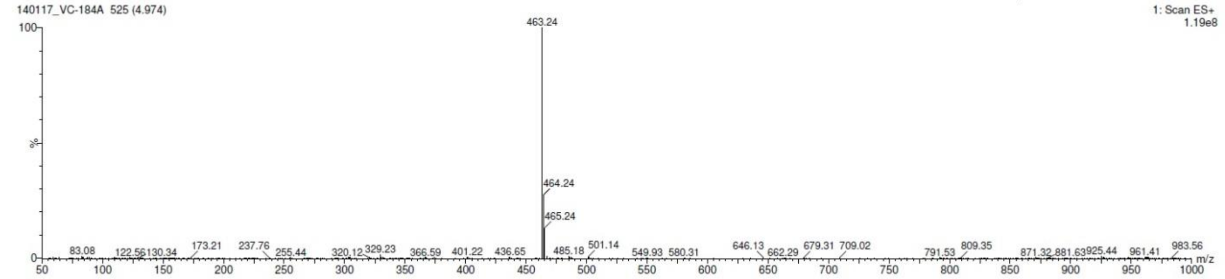

^{1}H-NMR 300 MHz, $CDCl_3$

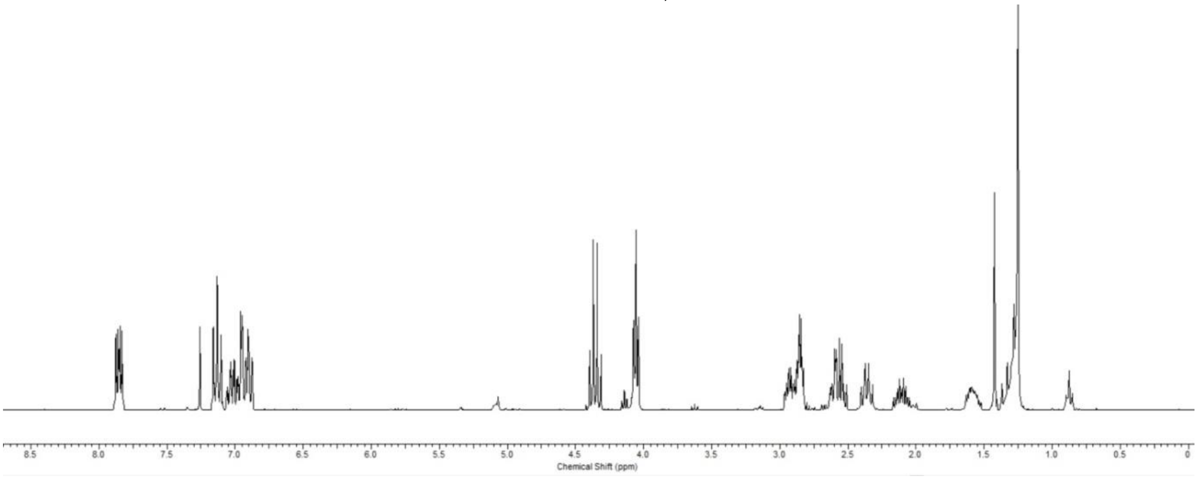

^{13}C-NMR 75 MHz, $CDCl_3$

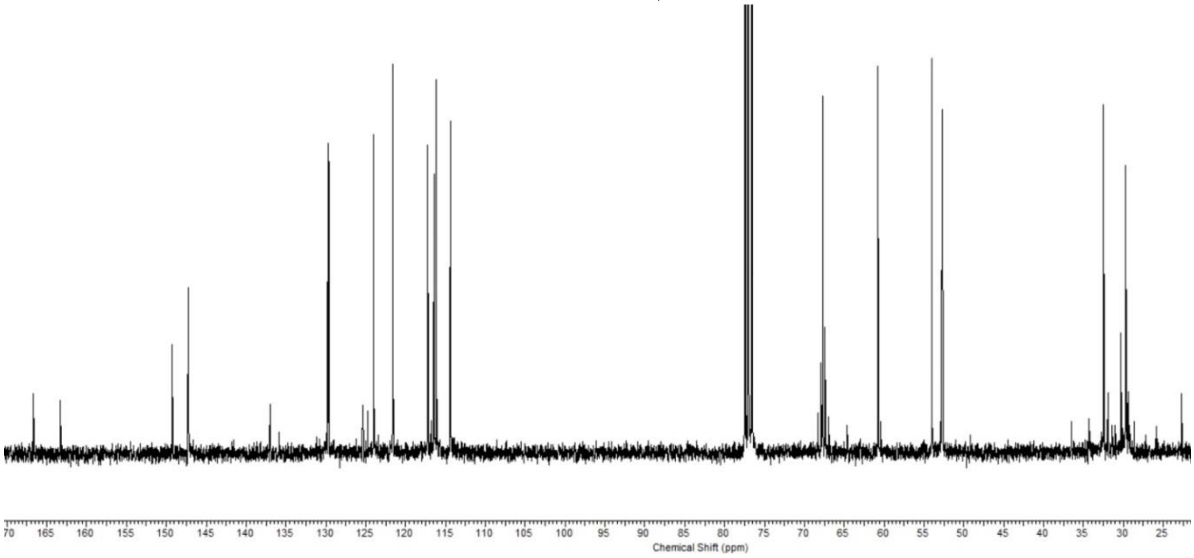

1.4. *(S)-3,4-Dimethoxy-N-(1-{2-[(2,2,2-trifluoroethoxy)phenoxy]ethyl}pyrrolidin-3-yl)benzenesulfonamide* (**18**)

MS

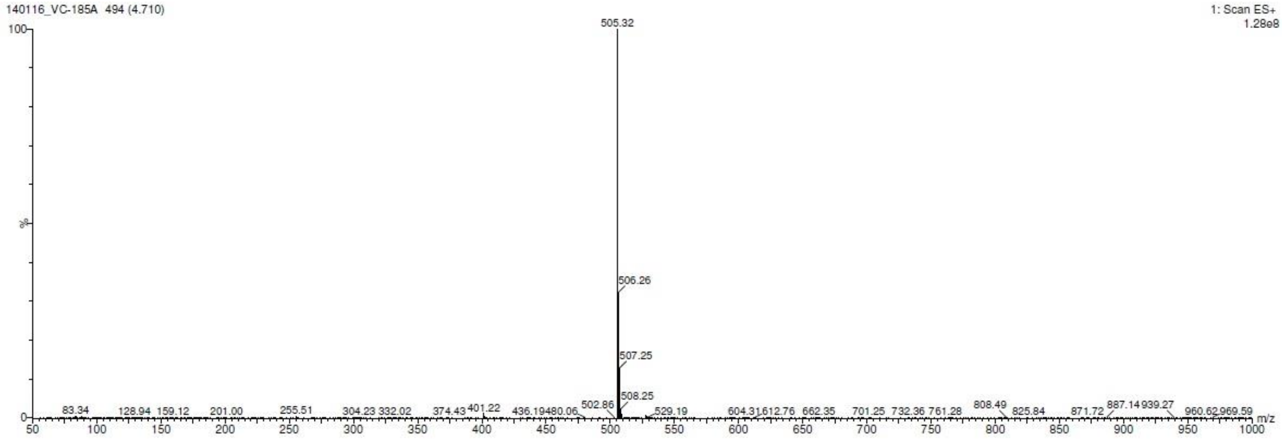

^{1}H-NMR 300 MHz, $CDCl_3$

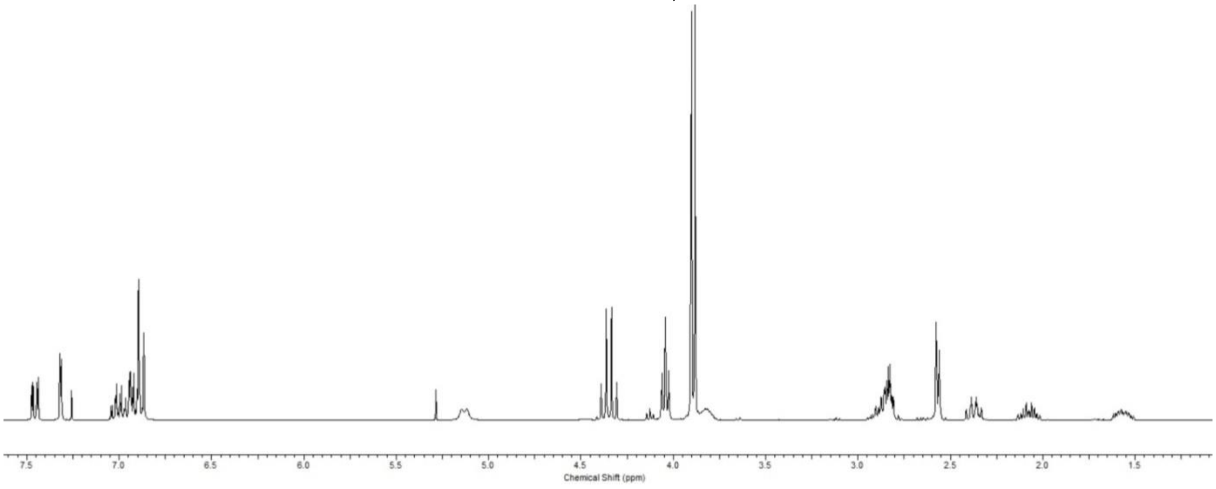

^{13}C-NMR 75 MHz, $CDCl_3$

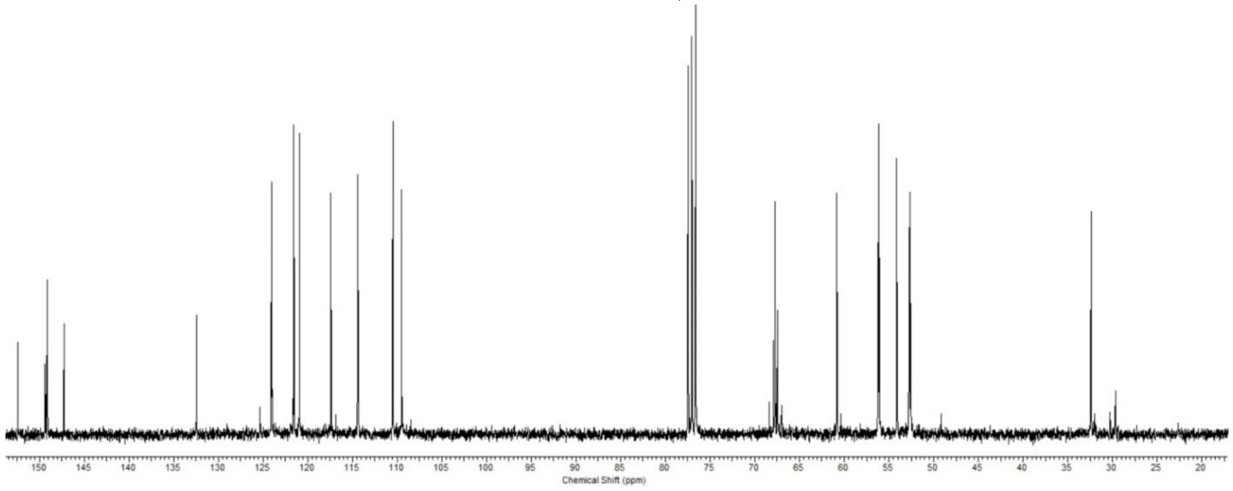

Supplement: Supplementary file 1 [file molecules-23-02175-s001.pdf]
